# Biphasic Regulation of p38 MAPK by Serotonin Contributes to the Efficacy of Stimulus Protocols That Induce Long-Term Synaptic Facilitation

**DOI:** 10.1523/ENEURO.0373-16.2017

**Published:** 2017-02-14

**Authors:** Yili Zhang, Paul Smolen, Douglas A. Baxter, John H. Byrne

**Affiliations:** Department of Neurobiology and Anatomy, W.M. Keck Center for the Neurobiology of Learning and Memory, McGovern Medical School, University of Texas Health Science Center at Houston, Houston, TX 77030

**Keywords:** biphasic regulation, computational model, ERK, LTF, p38 MAPK, spaced stimulation

## Abstract

The MAPK isoforms ERK and p38 MAPK are believed to play opposing roles in long-term synaptic facilitation (LTF) induced by serotonin (5-HT) in *Aplysia*. To fully understand their roles, however, it is necessary to consider the dynamics of ERK and p38 MAPK activation. Previous studies determined that activation of ERK occurred ∼45 min after a 5-min pulse of 5-HT treatment. The dynamics of p38 MAPK activation following 5-HT are yet to be elucidated. Here, the activity of p38 MAPK was examined at different times after 5-HT, and the interaction between the ERK and p38 MAPK pathways was investigated. A 5-min pulse of 5-HT induced a transient inhibition of p38 MAPK, followed by a delayed activation between 25 and 45 min. This activation was blocked by a MAPK kinase inhibitor, suggesting that similar pathways are involved in activation of ERK and p38 MAPK. ERK activity decreased shortly after the activation of p38 MAPK. A p38 MAPK inhibitor blocked this decrease in ERK activity, suggesting a causal relationship. The p38 MAPK activity ∼45 min after different stimulus protocols was also characterized. These data were incorporated into a computational model for the induction of LTF. Simulations and empirical data suggest that p38 MAPK, together with ERK, contributes to the efficacy of spaced stimulus protocols to induce LTF, a correlate of long-term memory (LTM). For example, decreased p38 MAPK activity ∼45 min after the first of two sensitizing stimuli might be an important determinant of an optimal interstimulus interval (ISI) for LTF induction.

## Significance Statement

MAPK pathways play critical roles in mediating diverse forms of synaptic plasticity. Activation of the ERK isoform is required for long-term synaptic facilitation (LTF), whereas the p38 MAPK isoform is required for long-term synaptic depression (LTD). Here, we used isolated *Aplysia* sensory neurons (SNs) to confirm and extend previous studies delineating dynamics of ERK and p38 MAPK. We quantified p38 MAPK activity after application of the essential neurotransmitter 5-HT and explored the cross talk between p38 MAPK and ERK in SNs. These data were incorporated into a computational model for the induction of LTF. Simulations suggest that p38 MAPK, together with ERK, contributes to the efficacy of temporally spaced stimulus protocols to induce LTF, a correlate of long-term memory (LTM).

## Introduction

MAPK pathways play critical roles in mediating diverse forms of synaptic plasticity (English and Sweatt, 1997; Adams and Sweatt, 2002; Hu et al., 2004). The interactions among MAPK isoforms are believed to regulate the nature (increases vs decreases) and duration (short- vs long-term) changes in synaptic strength. For example, the sensorimotor synapse in *Aplysia* exhibits at least two forms of long-term plasticity. Long-term synaptic facilitation (LTF) can be induced by repeated application of the neuromodulator serotonin (5-HT) (Schacher et al., 1990, 2000), whereas long-term synaptic depression (LTD) can be induced by application of the peptide Phe-Met-Arg-Phe-NH2 (FMRFa) (Montarolo et al., 1988; Schacher et al., 2000; Guan et al., 2002). Activation of the ERK isoform is required for LTF (Martin et al., 1997; Michael et al., 1998; Purcell et al., 2003; Ormond et al., 2004; Sharma and Carew, 2004), whereas activation of the p38 kinase isoform is required for LTD (Guan et al., 2002, 2003). What is less clear, however, are the ways in which these two opposing MAPK pathways are regulated and how interactions between the ERK and p38 kinase pathways lead to a specific outcome (e.g., LTF vs LTD). Guan et al. (2002, 2003) proposed a model in which the two pathways are regulated by different inputs and reciprocally inhibit one another. For example, they suggested that 5-HT-induced activation of protein kinase A (PKA), which is necessary for LTF, may in turn inhibit p38 MAPK, thereby further supporting LTF. Here, we investigate an alternative but not mutually exclusive hypothesis in which dynamics of ERK and p38 MAPK activation determine the outcome of plasticity mediated by MAPK pathways. 5-HT activation of ERK leads to inactivation of the transcription repressor CREB2 (Bartsch et al., 1995). It is likely that ERK also activates the transcription activator CREB1, via activation of the CREB kinase denoted ribosomal S6 kinase (Choi et al., 2011; Philips et al., 2013b). In contrast, p38 MAPK mediates LTD by activating CREB2 and enhancing CREB2-mediated repression of genes such as *c/ebp* (Guan et al., 2002; 2003). Moreover, the 5-HT and FMRFa pathways interact at the levels of ERK and p38 MAPK. 5-HT inhibits p38 MAPK activity and activates ERK, whereas FMRFa activates p38 MAPK and inhibits ERK (Guan et al., 2003; Fioravante et al., 2006). Consequently, a plausible hypothesis is that the dynamic balance of these MAPK isoforms determines the direction of synaptic plasticity.

This hypothesis is based, in part, on previous observations that the dynamics of ERK activation are complex (Ye et al., 2008; Philips et al., 2013b; see also Ajay and Bhalla, 2004). For example, in sensorimotor synapses, a single 5-min pulse of 50 μM 5-HT has no effect on ERK phosphorylation and, thus, activation when examined immediately after treatment. However, the 5-HT pulse induces a delayed activation of ERK ∼45 min after treatment. In contrast, Guan et al. (2003) reported that phosphorylation and thus activation of p38 MAPK is reduced immediately after a 10-min treatment of 50 μM 5-HT. Currently, nothing is known about the subsequent time course of p38 MAPK phosphorylation. Philips et al. (2013b) also found that ERK activity, elevated at 45 min, returns to control level at 60 min after a 5-min pulse of 50 μM 5-HT. It is not evident how ERK activity can be downregulated to control level within ∼15 min.

Here, we used isolated *Aplysia* sensory neurons (SNs) to confirm and extend the previous studies in dynamics of ERK and p38 MAPK. We quantified the dynamics of MAPK activity after a 5-min pulse of 5-HT (50 μM) and explored the cross talk between p38 MAPK and ERK pathways in SNs.

It is commonly accepted that spaced stimulus protocols (i.e., with long intervals between sessions) are more efficient in inducing LTF and long-term memory (LTM) than are massed protocols (i.e., short or no intervals between sessions) (Mauelshagen et al., 1998; Philips et al., 2013a; Smolen et al., 2016). Philips et al. (2007; 2013b) similarly found that two tail shocks to *Aplysia* could induce long-term sensitization of a withdrawal reflex, a form of LTM, but only when separated by 45 min. No LTM was observed with an interstimulus interval (ISI) that was too short (15 min) or too long (60 min). Simulations with a computational model we developed suggest that the ISI of 45 min is superior in inducing LTM, because the ratio of ERK to p38 MAPK activity is maximized by an ISI of ∼45 min, mainly due to a delayed decrease in p38 MAPK activity at this time.

## Materials and Methods

### Neuronal cultures

This study used juvenile *Aplysia*, which are hermaphroditic invertebrates. *Aplysia californica* (80-100 g) were supplied by The University of Miami and maintained in circulating artificial seawater at 15°C. SNs were isolated from the ventral-caudal cluster of the pleural ganglion from *Aplysia*. Before extraction of ganglia, animals were anesthetized by injection of isotonic MgCl_2_ equal to approximately one half of the body volume. Ganglia were removed and incubated in culture medium containing 50% hemolymph and 50% isotonic L15 at room temperature and then manually desheathed. SNs were removed from the ganglia individually by microelectrodes with fine tips and plated on poly-L-lysine-coated glass slides in petri dishes of culture medium. Dishes of SN cultures were plated with four to eight SNs and allowed to grow for 5 d at 18°C before experiments were begun.

### Pharmacological treatment

Dishes of SNs cultured from the same animals were paired for all the 5-HT treatments. In each pair, one dish received solution which consisted of 50% isotonic L15 and 50% artificial seawater (L15/ASW) (ASW: 450 mM NaCl, 10 mM KCl, 11 mM CaCl_2_, 29 mM MgCl_2_, and 10 mM HEPES, pH 7.6) as vehicle control (Veh). The other received the same solution with the addition of 50 μM 5-HT. The experimenter was blind to the identity of the treatments. In the experiments to measure the time course of phosphorylated ERK (pERK) or phosphorylated p38 MAPK (p-p38 MAPK) after one pulse of 5-HT, if four pairs of dishes were available, one dish was fixed immediately after wash off of 5-HT for immunofluorescence (see below). The other three dishes were incubated in L15/ASW after wash off of 5-HT, until they were fixed at 15, 45, and 60 min after onset of 5-HT for immunofluorescence. The remaining four dishes served as time-matched Veh controls. For each pair of dishes measured at the same time point, the averaged pERK or p-p38 MAPK from the dish receiving 5-HT was compared with the averaged pERK or p-p38 MAPK from the Veh control. If two to three pairs were available, one pair was fixed immediately after wash off of 5-HT, the others were fixed at one or two of the above later times. When two pulses of 5-HT were given to SNs, the ISI was defined as the interval from the onset of the first stimulus to the onset of the second stimulus. During the ISI, SNs were incubated in L15/ASW after wash out of 5-HT.

To examine the effects of p-p38 MAPK on pERK, 3 μM SB203580 (EMD Millipore) was applied to SN cultures 30 min before 5-HT treatments (one 5-min pulse of 50 μM 5-HT) and then concurrently with 5 min 5-HT treatment. At this concentration (3 μM) SB203580 blocks LTD in *Aplysia* without affecting basal synaptic strength (Guan et al., 2003). Four dishes of SNs from the same animals were used for each experiment. Each dish was given a different treatment, either (1) 50 μM 5-HT alone, (2) 3 μM SB203580 alone, (3) 5-HT + SB203580, or (4) Veh alone. After 5-HT was washed out, treatment with SB203580 was continued until fixation for immunofluorescence.

To examine the effect of MEK1/2 inhibition on p-p38 MAPK, 20 μM U0126 (Promega) was applied to SN cultures 30 min before and then concurrently with 5-min 50 μM 5-HT treatment. At this concentration (20 μM), U0126 blocks ERK phosphorylation after 5-HT treatment in *Aplysia* (Sharma et al., 2003). Four dishes of SNs from the same animals were used for each experiment and given different treatments, either (1) 50 μM 5-HT alone, (2) 20 μM U0126 alone, (3) 5-HT + U0126, or (4) Veh alone. After 5-HT was washed out, U0126 treatment was continued until fixation for immunofluorescence.

### Immunofluorescence

Immunofluorescence procedures for SNs followed those of Liu et al. (2014). Briefly, at different time points after the onset of 5-HT treatment, cells were fixed in a solution of 4% paraformaldehyde in PBS containing 20% sucrose. After three 5-min rinses in PBS, fixed cells were blocked for 30 min at room temperature in a solution of Superblock buffer (Pierce), 0.2% Triton X-100 and 3% normal goat serum, and subsequently incubated overnight at 4°C with anti-pERK rabbit antibody (1:200), or anti-p-p38 MAPK rabbit antibody (1:200). Anti-pERK antibodies [phospho-p44/42 MAPK (Erk1/2) XP Rabbit mAb, catalog #4370S; RRID: AB2315112] and anti-p-p38 MAPK antibodies [phospho-p38 MAPK (Thr180/Tyr182) XP Rabbit mAb, catalog #4511S; RRID: AB2139682] were purchased from Cell Signaling Technology. Secondary antibody (goat anti-rabbit secondary antibody conjugated to Rhodamine Red; 1:200 dilution; Thermo Scientific, catalog #R-6394) was applied for 1 h at room temperature. Cells were then mounted using Mowiol 4-88 (Sigma-Aldrich). The intensity of staining in SNs was quantified in images obtained with a Zeiss LSM510 confocal microscope using a 63× oil-immersion lens as described previously (Liu et al., 2014). A *z*-series of optical sections through the cell body (0.5-μm increments) were taken, and the section through the middle of the nucleus was used for analysis of mean fluorescence intensity with MetaMorph Offline software (Universal Imaging). Four to eight neurons on each coverslip were analyzed, and measurements from neurons on the same coverslip were averaged.

### Statistical analyses

SigmaPlot version 11 (Systat Software) was used. Data are presented by box-and-whisker plots, and *p <* 0.05 was considered to represent statistical significance. Before making comparisons between groups, Shapiro--Wilk normality and equal variance tests were performed. For data displaying normal distribution, paired *t* test was used for comparison between paired Veh and treatment groups, and one-way ANOVA and the *post hoc* Student--Newman--Keuls method were used for multiple comparisons between groups. For data displaying non-normal distribution, Wilcoxon signed-rank test (WSRT) was used for comparison between paired Veh and treatment groups, and Kruskal--Wallis one-way ANOVA on ranks and the *post hoc* Student--Newman–Keuls method were used for multiple comparisons between groups.

## Model development and equations

The mathematical model for the activation (i.e., phosphorylation) of ERK and p38 MAPK was modified from a previously published model (Zhang et al., 2012) of the signaling cascades underlying the induction of LTF.

### ERK pathway

The activation of ERK was modeled as a cascade with sequential activation of the kinases Raf, MEK1 (MAPKK), and ERK. The ordinary differential equations describing the activation or phosphorylation of Raf, MEK1, and ERK (Eqs. 1-8) are similar to those in Zhang et al. (2012). To allow simulation of the quantitative changes of pERK by 5-HT, basal kinase activities were added to the equations describing the Raf pathway (Eq. 1). [*MEK1*] in Eqs. 3-10 represents the MEK1/2 isoforms known to phosphorylate ERK (Shaul and Seger, 2007). [*ERK^pp^*] corresponds to the pERK level measured by immunofluorescence. As described in Zhang et al. (2012), a discrete time delay (τ_delay_ = 25 min) was added to the phosphorylation of Raf in Eq. 1. Because the mechanism underlying the delay in ERK phosphorylation is unknown, τ_delay_ is used to ensure that pERK has a peak at ∼45 min after onset of 5-HT.

(Eq. 1) $$\frac{d[Raf^{p}]}{dt}=(k_{basal,Raf}+k_{f,Raf}{〈\left[5\text{-}HT\right]〉}_{\tau_{delay}})[Raf]-k_{b,Raf}[Raf^{p}]$$

(Eq. 2) $$\left[Raf]={\left[Raf\right]}_{total}-[Raf^{p}\right]$$

(Eq. 3) $$\frac{d[MEK1]}{dt}=\frac{k_{b,MEK}[MEK1^{p}]}{[MEK1^{p}]+K_{MEK,2}}-\frac{k_{f,MEK}[Raf^{p}][MEK1]}{[MEK1]+K_{MEK,1}}$$

(Eq. 4) $$\frac{d[MEK1^{pp}]}{dt}=\frac{k_{f,MEK}[Raf^{p}][MEK1^{p}]}{[MEK1^{p}]+K_{MEK,1}}-\frac{k_{b,MEK}[MEK1^{pp}]}{[MEK1^{pp}]+K_{MEK,2}}$$

(Eq. 5) $$\left[MEK1^{p}]={\left[MEK1\right]}_{total}-[MEK1]-[MEK1^{pp}\right]$$

(Eq. 6) $$\frac{d[ERK]}{dt}=\frac{k_{b,ERK}[ERK^{p}]}{[ERK^{p}]+K_{ERK,2}}-\frac{k_{f,ERK}[MEK1^{pp}][ERK]}{[ERK]+K_{ERK,1}}$$

(Eq. 7) $$\frac{d[ERK^{pp}]}{dt}=\frac{k_{f,ERK}[MEK1^{pp}][ERK^{p}]}{[ERK^{p}]+K_{ERK,1}}-\frac{k_{b,ERK}[ERK^{pp}]}{[ERK^{pp}]+K_{ERK,2}}$$

(Eq. 8) $$\left[ERK^{p}]={\left[ERK\right]}_{total}-[ERK]-[ERK^{pp}\right]$$

We found that p-p38 MAPK inhibits ERK activation (see Results). The mechanism of this inhibition is not yet determined. Two plausible hypotheses are that activated p38 MAPK enhances MEK1 dephosphorylation, or enhances ERK dephosphorylation, in both cases indirectly via activation of an undetermined phosphatase. In the absence of discriminating data, we simulated the former mechanism. Thus, the dephosphorylation rate of MEK1, *k_b,MEK_*, was assumed to be enhanced by p-p38 MAPK (Eqs. 9, 10). *E_p38_* represents this inhibitory effect of p-p38 MAPK. The Heaviside step function *heav([MEK1^pp^] − [MEK1^pp^]_basal_)* is 0 if MEK1 is equal to or lower than the basal level (i.e., *[MEK1^pp^]_basal_*) and is 1 if MEK1 is larger than the basal level. This function is used to support the empirical finding that p-p38 MAPK has no effect on the basal level of MEK1/2 (see Results).

(Eq. 9) $$k_{b,MEK}=k_{b,MEK\_basal}+k_{b,MEK\_p38}E_{p38}$$

(Eq. 10) $$\frac{d[E_{p38}]}{dt}=k_{EP38}[P38^{pp}]heav([MEK1^{pp}]-{\left[MEK1^{pp}\right]}_{basal})-k_{d,EP38}E_{p38}$$

### p38 MAPK pathway

The mechanism underlying the inhibition and activation of p38 MAPK by 5-HT has yet to be characterized. Based on the empirical results of this study, we assumed that 5-HT engages two separate pathways to, respectively, inhibit and activate p38 MAPK. In the first pathway, 5-HT inhibits the phosphorylation rate (*k_f,p38_*) of p38 MAPK (Eqs. 19, 20). In the second pathway, a delayed phosphorylation of p38 MAPK due to 5-HT occurs via a p38 MAPK-specific-MAPKK kinase (MAPKKK)/MAPKK cascade (Eqs. 11-18) similar to the one that phosphorylates ERK; however, it is still unknown which isoform(s) of MAPKK phosphorylate p38 MAPK in *Aplysia*. *[MAPKK_p38_]* represents this unknown MAPKK isoform. The differential equations describing the p38 MAPK pathway are,

(Eq. 11) $$\frac{d[MAPKKK_{p38}^{p}]}{dt}=(k_{basal,Rafp38}+k_{f,Rafp38}[5-HT])\times [MAPKKK_{p38}] -k_{b,Rafp38}[MAPKKK_{p38}^{p}]$$

(Eq. 12) $$\left[MAPKKK_{p38}]={\left[MAPKKK_{p38}\right]}_{total}-[MAPKKK_{p38}^{p}\right]$$

(Eq. 13) $$\frac{d[MAPKK_{p38}]}{dt}=\frac{k_{b,MAPKK}[MAPKK_{{}^{p38}}^{p}]}{[MAPKK_{{}^{p38}}^{p}]+K_{MAPKK,2}}-\frac{k_{f,MAPKK}[MAPKKK_{{}^{p38}}^{p}][MAPKK_{p38}]}{[MAPKK_{p38}]+K_{MAPKK,1}}$$

(Eq. 14) $$\frac{d[MAPKK_{{}^{p38}}^{pp}]}{dt}=\frac{k_{f,MAPKK}[MAPKKK_{{}^{p38}}^{p}][MAPKK_{{}^{p38}}^{p}]}{[MAPKK_{{}^{p38}}^{p}]+K_{MAPKK,1}}-\frac{k_{b,MAPKK}[MAPKK_{{}^{p38}}^{pp}]}{[MAPKK_{{}^{p38}}^{pp}]+K_{MAPKK,2}}$$

(Eq. 15) $$\left[MAPKK_{{}^{p38}}^{p}]={\left[MAPKK_{p38}\right]}_{total}-[MAPKK_{p38}]-[MAPKK_{{}^{p38}}^{pp}\right]$$

(Eq. 16) $$\frac{d[P38]}{dt}=\frac{k_{b,p38}[P38^{p}]}{[P38^{p}]+K_{p38,2}}-\frac{k_{f,p38}[MAPKK_{p38}^{pp}][P38]}{[P38]+K_{p38,1}}$$

(Eq. 17) $$\frac{d[p38^{pp}]}{dt}=\frac{k_{f,p38}[MAPKK_{p38}^{pp}][p38^{p}]}{[p38^{p}]+K_{p38,1}}-\frac{k_{b,p38}[p38^{pp}]}{[p38^{pp}]+K_{p38,2}}$$

(Eq. 18) $$\left[p38^{p}]={\left[p38\right]}_{total}-[p38]-[p38^{pp}\right]$$

(Eq. 19) $$k_{f,p38}=\frac{k_{f,p38\_max}}{1+E_{5-HT}}$$

(Eq. 20) $$\frac{d[E_{5-HT}]}{dt}=k_{E5HT}\frac{\left[5-HT\right]}{[5-HT]+K_{5HT\_p38}}-k_{d,E5HT}E_{5-HT}$$

*[p38^pp^]* in Eq. 17 corresponds to the p-p38 MAPK level measured by immunofluorescence. The parameters of the p38 MAPK cascade were adjusted so that a pulse of 5-min 5-HT elicited a transient decrease, followed by a delayed increase, of p-p38 MAPK (see Results).

The standard parameter values for the ERK and p38 MAPK pathway model are:*k_basal,Raf_*= 0.006 min^−1^, *k_f,Raf_*= 0.0023 μM^−1^min^−1^, *k_b,Raf_*= 0.017 min^−1^, *[Raf]_total_*= 0.5 μM, *k_f,MEK_*= 0.41 min^−1^, *k_b,MEK_basal_*= 0.04 μM/min, *k_b,MEK_p38_*= 0.04 min^−1^, *K_MEK,1_*= 0.20 μM, *K_MEK,2_*= 0.19 μM, *[MEK1]_total_*= 0.5 μM, *k_f,ERK_*= 0.41 min^−1^, *k_b,ERK_*= 0.12 μM/min, *K_ERK,1_*= 0.19 μM, *K_ERK,2_*= 0.21 μM, *[ERK]_tota_*_l_ = 0.5 μM, *k_EP38_* = 0.5 μM^−1^ min^−1^, *[MEK1^pp^]_basal_* = 0.26 μM, *k_d,EP38_* = 0.1 min^−1^, *k_basal,Rafp38_*= 0.029 min^−1^, *k_f,Rafp38_*= 0.003 μM^−1^min^−1^, *k_b,Rafp38_*= 0.08 min^−1^, *[Raf_p38_]_total_*= 0.5 μM, *k_f, MAPKK_* = 0.41 min^−1^, *k_b,MAPKK_*= 0.04 μM/min, *K _MAPKK,1_*= 0.20 μM, *K _MAPKK,2_*= 0.19 μM, *[MAPKK _p38_]_total_*= 0.5 μM, *k_f,p38_max_*= 0.41 min^−1^, *k_b,p38_*= 0.12 μM/min, *K_p38,1_*= 0.19 μM, *K_p38,2_*= 0.21 μM, *[p38]_tota_*_l_ = 0.5 μM, *k_E5HT_*= 0.25 min^−1^, *K_5HT_p38_*= 50 μM, *k_d,E5_HT_* = 0.1 min^−1^.

### Numerical methods

Fourth-order Runge--Kutta integration was used for integration of differential equations with a time step of 3 s. No significant improvement in accuracy was found upon further time step reduction. Prior to any stimuli, the steady-state levels of variables were determined after at least two simulated days. The model was programmed in XPPAUT (RRID: SCR001996) and simulated on Dell precision T1700 computers. Source codes will be submitted to ModelDB (McDougal et al., 2015).

## Results

### 5-HT induced an initial decrease in p-p38 MAPK followed by an increase

**Figure 1.**
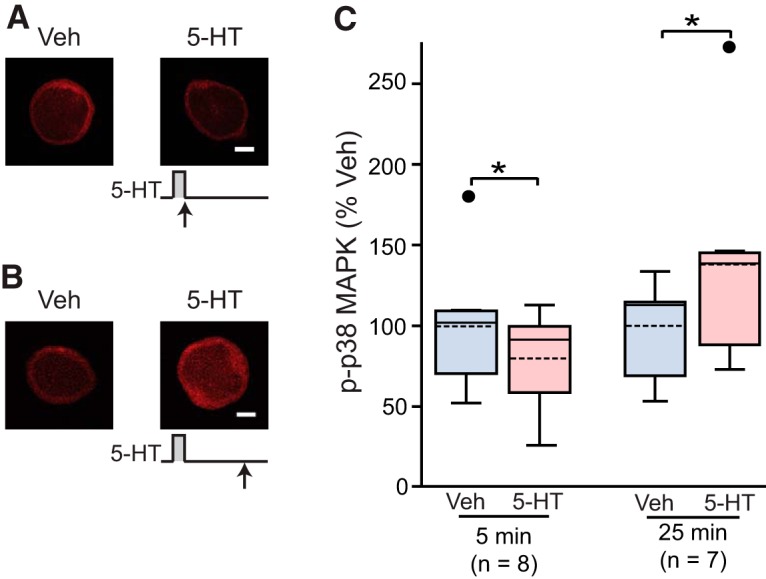
p-p38 MAPK at different times after one 5-min pulse of 5-HT (50 μM). ***A***, Representative confocal images of p-p38 MAPK immunofluorescence in SNs immediately after the end of 5-HT. ***B***, Representative confocal images of p-p38 MAPK immunofluorescence in SNs at 25 min after onset of 5-HT. ***C***, Summary data. p-p38 MAPK levels were normalized to the mean of Veh controls. Treatment with 5-HT for 5 min significantly decreased p-p38 MAPK immediately after the end of treatment, whereas the same treatment led a significant increase in p-p38 MAPK when measured 25 min after onset of 5-HT. In this and other illustrations, data are presented by box-and-whisker plots. The median is indicated by the solid line in the interior of the box. The mean is indicated by the dashed line in the interior of the box. The lower end of the box is the first quartile (Q1). The upper end of the box is the third quartile (Q3). The ends of the vertical lines (whiskers) are the maximum and minimum values of nonoutliers. The small circles outside the whiskers are the outliers larger than Q3 + 1.5(Q3-Q1) or smaller than Q3-1.5(Q3-Q1). Scale bar, 20 μm. Significant differences are indicated by * for *p* < 0.05.

MAPK isoforms are activated by phosphorylation of the threonine and tyrosine residues in their activation loops. Thus, in this study, the levels of pERK and of p-p38 MAPK are used as measures of ERK and p38 MAPK activity. A 10-min treatment of 50 μM 5-HT to *Aplysia in vivo* produces a ∼30% reduction in p-p38 MAPK in *Aplysia* pleural ganglia as measured with Western blot (Guan et al., 2003). However, it is not known whether a similar decrease is produced in individual SNs, nor have the dynamics of p-p38 MAPK at later time points been examined. We used immunofluorescence to quantify p-p38 MAPK in isolated SNs immediately or at a later time after treatment. Two treatment groups were examined: (1) one pulse of 5 min 5-HT, with fixation immediately after treatment ended; and (2) one pulse of 5 min 5-HT, fixed at 25 min after onset of 5-HT. Example responses are illustrated in Figure 1*A,B*, and summary data are in Figure 1*C*. 5-HT led to a 20 ± 6.2% (*n* = 8; *n* here and also in other experiments represents the number of dishes) decrease in p-p38 MAPK levels measured immediately after treatment (WSRT, *Z* = 2.521; *p* = 0.008)^a^ (superscript letters a–i refer to entries in the statistical table, see Table 1). These results indicate the 5-HT-induced decrease in p-p38 MAPK observed in ganglia by Guan et al. (2003) are also observed in individual SNs. We also found that the initial decrease was followed by a 39 ± 17% (*n* = 7) increase in p-p38 MAPK above control, at 25 min after the 5-min treatment (WSRT, *Z* = 2.197; *p* = 0.03)^b^.

**Table 1:** Statistical Table

	**Data structure**	**Type of test**	**Power**
a	Non-normal distribution	Wilcoxon signed-rank test	95% confidence interval of the difference: -20.6 to -1.2
b	Non-normal distribution	Wilcoxon signed-rank test	95% confidence interval of the difference: -4.6 to 35.2
c	Non-normal distribution	Wilcoxon signed-rank test	95% confidence interval of the difference: -16.7 to -0.4
d	Normal distribution	Paired *t* test	Power: 0.956
e	Normal distribution	Paired *t* test	Power: 0.992
f	Normal distribution	One-way RM ANOVA + Student--Newman--Keuls	Power: 0.858
g	Normal distribution	One-way RM ANOVA + Student--Newman--Keuls	Power: 0.805
h	Normal distribution	One-way ANOVA	Power: 0.066
i	Non-normal distribution	Kruskal--Wallis one-way ANOVA on ranks + Student--Newman--Keuls	Not applicable

**Figure 2.**
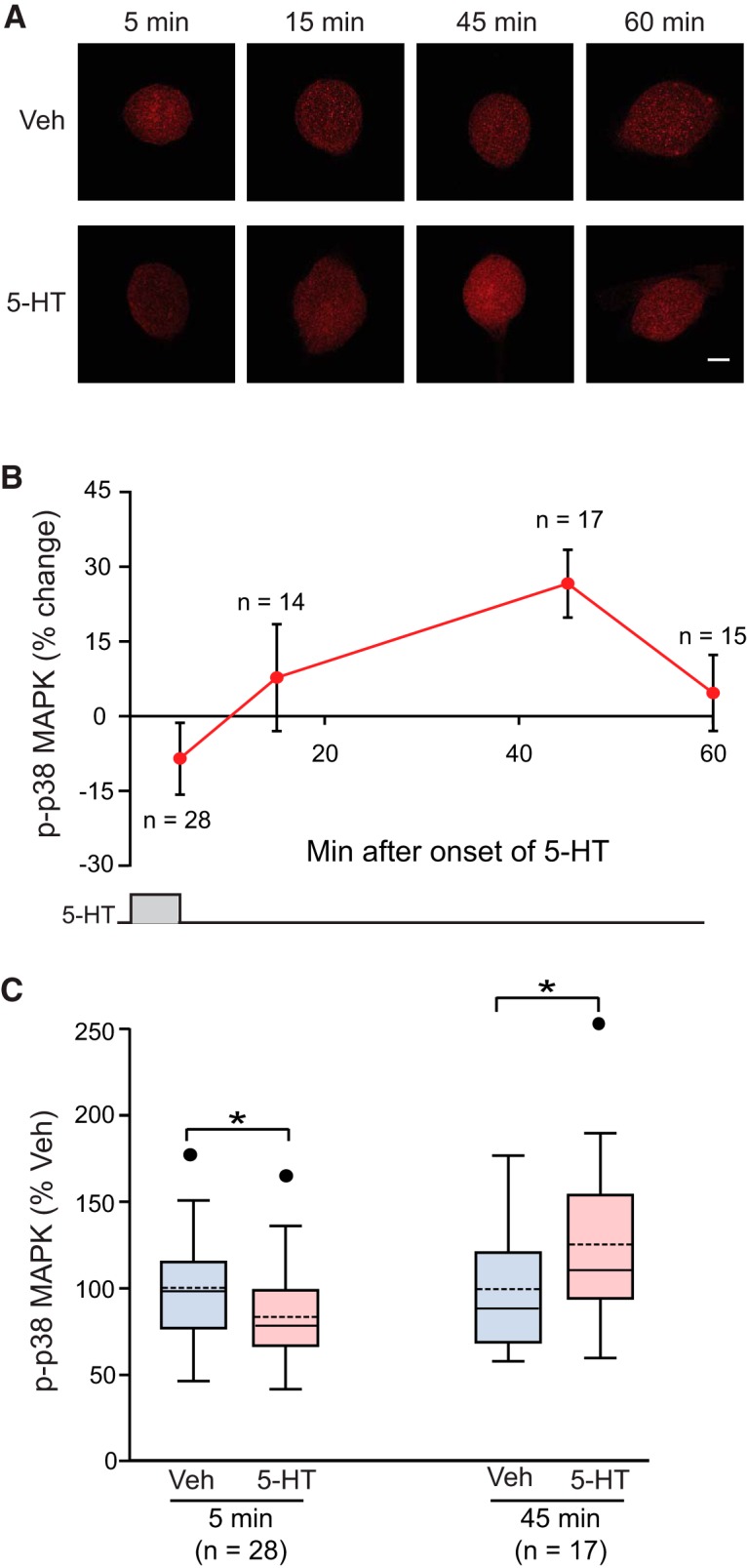
Biphasic regulation of p38 MAPK by one 5-min pulse of 5-HT (50 μM). ***A***, Representative confocal images of p-p38 MAPK immunofluorescence in *Aplysia* SNs at different times after onset of 5-HT. Scale bar, 20 μm. ***B***, Summary data. The percent change was calculated as the change of p-p38 MAPK level after 5-HT compared with control level. 5-HT induced a delayed increase in p-p38 MAPK, following a transient decrease immediately after treatment. p-p38 MAPK returned to the control level at 60 min. ***C***, Statistical analysis revealed significant differences between Veh and 5-HT treatment groups at 5 and 45 min. p-p38 MAPK levels were normalized to the mean of Veh controls. Significant differences between the groups are indicated by * for *p* < 0.05.

To explore in more detail the time course of p38 MAPK after one pulse of 5-HT, immunofluorescence was used to quantify p-p38 MAPK in isolated SNs fixed at 5, 15, 45, and 60 min after onset of a 5-min pulse of 50 μM 5-HT. These time points and duration of 5-HT treatment correspond to those used to measure pERK (Philips et al., 2013b). Figure 2*A,B* illustrates that 5-HT led to an initial reduction (-8.6 ± 7.2%, *n* = 28) of p-p38 MAPK (the 5-min time point; see also Figure 1*C*). This initial decrease was followed by a return of p-p38 MAPK to near control level at 15 min (7.7 ± 10.7%, *n* = 14), an increase above control at 45 min (26.7 ± 6.8%, *n* = 17), and then a return to control level at 60 min (4.7 ± 7.6%, *n* = 15). Statistical analyses revealed that the effects at the 5- and 45-min time points (Fig. 2*C*) were statistically significant (5 min, WSRT, *Z* = 2.960; *p* = 0.003; 45 min, paired *t* test, *t*_(16)_ = 3.669; *p* = 0.002)^c,d^. These results indicate that a brief pulse of 5-HT leads to a biphasic regulation of p-38 MAPK, an initial decrease, followed by a late increase, and subsequent decline.

### p38 MAPK appears to mediate the decrease of pERK 60 min after 5-HT

What is the functional significance of the late increase in p-p38 MAPK? Treatment with 5-HT increases the levels of pERK at 45 min, which return to control level at 60 min (Philips et al., 2013b). The mechanism for this rapid drop is not known. One possibility is that the late increase in p-p38 MAPK (Fig. 2) produces a feed-forward late inhibition of MEK1/2 (Westermarck et al., 2001). If so, an inhibitor of p-p38 MAPK should boost pERK 60 min after treatment. The increase of pERK at 45 min was confirmed with immunofluorescence techniques in this study (data not shown). A 5-min treatment with 50 μM 5-HT led to a 48 ± 11% increase in pERK (*n* = 8, paired *t* test, *t*_(7)_ = 5.112; *p* < 0.05)^e^.

**Figure 3.**
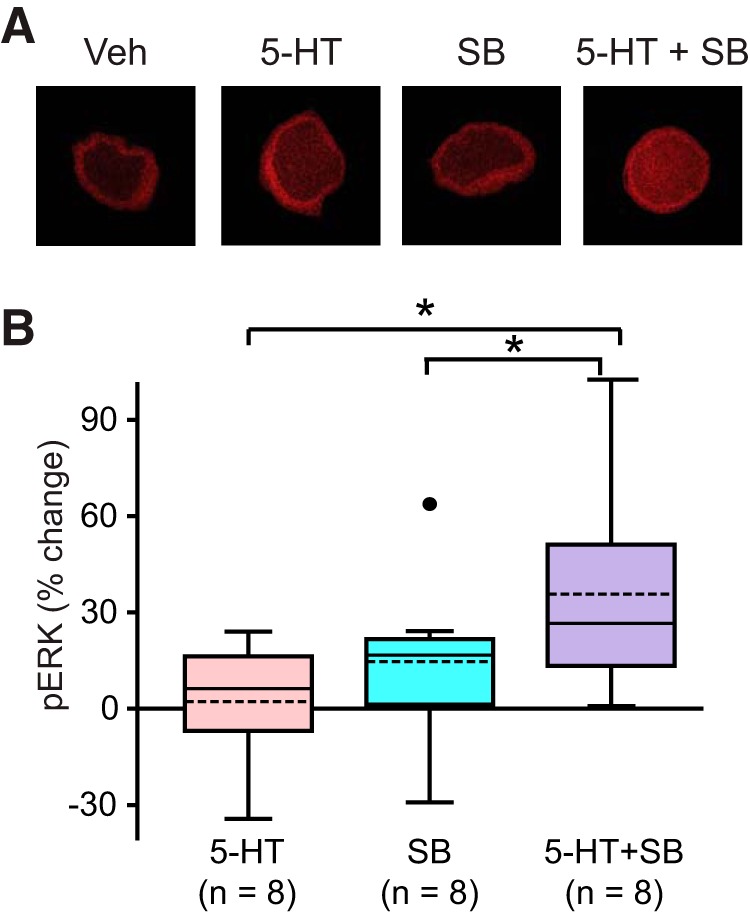
The effect of p38 MAPK inhibitor on pERK. ***A***, Representative confocal images of pERK immunofluorescence in SNs at 60 min after onset of a 5-min pulse of 5-HT, in the absence or presence of the p38 MAPK inhibitor SB203580 (SB). ***B***, Summary data. Compared with the 5-HT alone group, treatment with 5-HT in the presence of SB induced a significant increase in pERK at 60 min after onset of 5-HT. Scale bar, 20 μm. Significant differences are indicated by * for *p* < 0.05.

Next, immunofluorescence was used to examine levels of pERK at 60 min after onset of 5-HT, a time point where pERK is expected to have returned to baseline (Philips et al., 2013b). Measurements of pERK were made in the absence or presence of the p38 MAPK inhibitor SB203580. SB203580 was applied to SNs 30 min before and during 5 min, 50 μM treatment with 5-HT. SNs continued to be treated with SB203580 until fixation at 60 min after onset of 5-HT. Four groups were examined: (1) 5-HT alone, (2) 3 μM SB203580 alone, (3) 5-HT + SB203580, and (4) Veh alone. Example responses are illustrated in Figure 3*A*, and summary data are presented in Figure 3*B*. Confirming Philips et al. (2013b), 5-HT alone had little effect (3 ± 6.3% change) on pERK at 60 min (*n* = 8). In contrast, 5-HT led to a 36 ± 11% (*n* = 8) increase in pERK in the presence of SB203580.

A one-way repeated measures (RM) ANOVA revealed a significant overall effect of the treatments (*F*_(3,21)_ = 5.968, *p* < 0.01)^f^. Subsequent pairwise comparisons (Student--Newman--Keuls) revealed that the 5-HT + SB203580 group was significantly different from the Veh group (*q* = 5.217, *p* < 0.01, *n* = 8)^f^, from the 5-HT alone group (*q* = 5.146, *p* < 0.01, *n* = 8)^f^, and from the SB203580 alone group (*q* = 3.381, *p* = 0.03, *n* = 8)^f^. No significant difference was detected between the 5-HT alone group and the Veh group (*q* = 0.0717, *p* = 0.96, *n* = 8)^f^, between the SB203580 alone group and the Veh group (*q* = 1.837, *p* = 0.41, *n* = 8)^f^, or between the 5-HT alone group and the SB203580 alone group (*q* = 1.765, *p* = 0.23, *n* = 8)^f^. These results support the hypothesis that p38 MAPK is involved in the decrease of pERK at 60 min after 5-HT, possibly via inhibition of a MEK pathway.

### MAPKK is involved in the delayed phosphorylation of p38 MAPK by 5-HT

The finding that the decrease of pERK 60 min after 5-HT results from an inhibitory effect of the p38 MAPK pathway raised a question whether the decrease of p38 MAPK 60 min after 5-HT results from a similar inhibitory effect of the ERK pathway. To explore the possibility of such mutual inhibition, immunofluorescence was used in pilot experiments (data not shown) to quantify p-p38 MAPK at 60 min after onset of 5-HT in the presence of the MEK1/2 inhibitor U0126. U0126 was applied to SNs 30 min before and during 5-HT. SNs continued to be treated with U0126 until fixation, 60 min after onset of 5-min 50 μM 5-HT. If the ERK pathway inhibits p38 MAPK activity, U0126 should relieve the inhibition and enhance p-p38 MAPK at 60 min after onset. Surprisingly, a small decrease of p-p38 MAPK by U0126 was observed. This result raised the intriguing possibility that U0126 may also attenuate the normal increase in p38 MAPK at 45 min after onset of 5-HT (Fig. 2).

**Figure 4.**
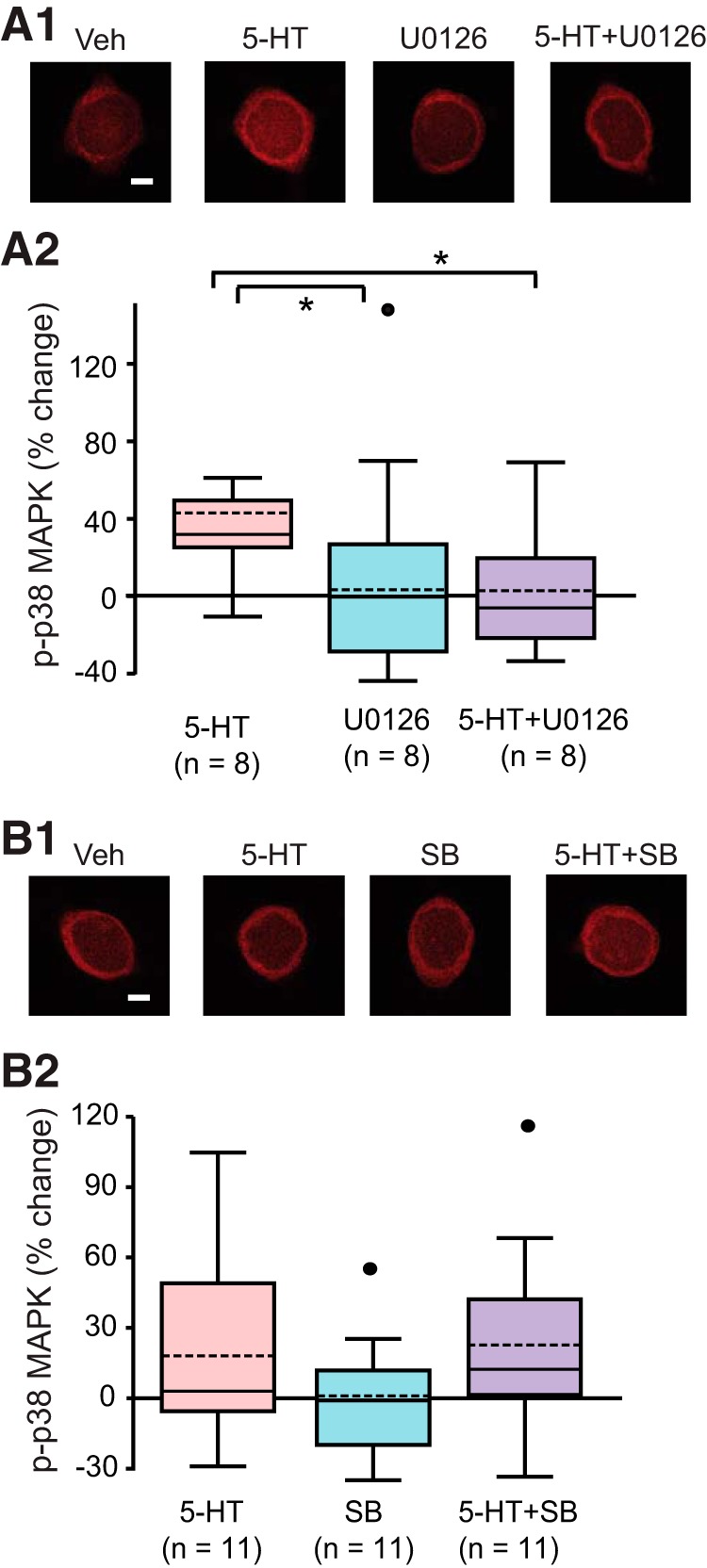
The interaction between p38 MAPK and MAPKK/ERK pathways. ***A1***, Representative confocal images of p-p38 MAPK immunofluorescence in SNs at 45 min after onset of 5-HT, in the absence or presence of the MEK1/2 inhibitor U0126. ***A2***, Summary data. Treatment with U0126 induced a significant decrease in 5-HT induced p-p38 MAPK at 45 min. ***B1***, Representative confocal images of p-p38 MAPK immunofluorescence in SNs at 60 min after onset of 5-HT, in the absence or presence of SB. ***B2***, Summary data. No significant differences were found among the three groups. Scale bar, 20 μm. Significant differences are indicated by * for *p* < 0.05.

To examine this possibility, U0126 was applied to SNs 30 min before and during 5-HT, and p-p38 MAPK was measured at 45 min after onset of 5-HT. SNs continued to be treated with U0126 until fixation, 45 min after onset of 5-min 50 μM 5-HT. Four groups were examined: (1) 5-HT alone, (2) 20 μM U0126 alone, (3) 5-HT + U0126, and (4) Veh alone. Example responses are illustrated in Figure 4*A1* , and summary data are presented in Figure 4*A2* . Replicating the results of Figure 2, 5-HT led to an increase (43 ± 15%) in levels of p-p38 MAPK (*n* = 8). In contrast, the increase was blocked (2.1 ± 11%) in the presence of U0126 (*n* = 8). A one-way RM ANOVA revealed a significant overall effect of the treatments (*F*_(3,21)_ = 5.383, *p* < 0.01, *n* = 8)^g^. Subsequent pairwise comparisons (Student--Newman--Keuls) revealed that the 5-HT alone group was significantly different from the Veh group (*q* = 4.045, *p* = 0.01, *n* = 8)^g^, from the 5-HT + U0126 group (*q* = 4.696, *p* < 0.01, *n* = 8)^g^, and from the U0126 alone group (*q* = 4.98, *p* = 0.01, *n* = 8)^g^. No significant difference was observed between the U0126 alone group and the Veh group (*q* = 0.935, *p* = 0.79, *n* = 8)^g^, between the 5-HT+ U0126 group and the Veh group (*q* = 0.652, *p* = 0.65, *n* = 8)^g^, or between the 5-HT+ U0126 group and the U0126 alone group (*q* = 0.284, *p* = 0.84)^g^. These results indicate that U0126 can suppress p-p38 MAPK elevation at 45 min after 5-HT onset, supporting the hypothesis that a p38 MAPK-specific-MAPKKK/MAPKK pathway is involved in the delayed phosphorylation of p38 MAPK by 5-HT. However, it is unclear which isoform of MAPKK is involved in the delayed phosphorylation of p38 MAPK in *Aplysia*. In mammalian cells, U0126 inhibits MEK1/2 but is not effective in inhibiting other MAPKK isoforms (e.g., MKK3 and MKK6) responsible for phosphorylation of p38 MAPK (Duncia et al., 1998). It is unclear whether U0126 is only effective in inhibiting MEK1/2 in *Aplysia*. It is possible that U0126 also inhibits a MAPKK isoform, distinct from MEK1/2, that activates p38 MAPK in *Aplysia*.

SB203580 inhibits downstream effects of p38 MAPK by preventing p-p38 MAPK from interacting with substrates (Cuenda and Rousseau, 2007). SB203580 was shown above to boost pERK at 60 min, plausibly through releasing the suppression of MEK by p-p38 MAPK. If the same MEK isoforms, MEK1/2, do in fact activate p38 MAPK directly or indirectly via pERK, then p-p38 MAPK will form an autoinhibitory feedback loop, via suppression of MEK1. 5-HT alone had little effect on pERK at 60 min after onset, but a 5-HT-induced increase in pERK was observed in the presence of SB203580 (Fig. 3). Thus, SB203580 might be expected to also increase p-p38 MAPK at 60 min after onset of 5-HT by breaking this inhibitory feedback loop. To investigate this hypothesis, immunofluorescence was used to quantify p-p38 MAPK in the presence of SB203580. SB203580 was applied to SNs 30 min before and during 5-HT. SNs continued to be treated with SB203580 until fixation, at 60 min after onset of 5-HT. Four groups were examined: (1) 50 μM 5-HT alone, (2) 3 μM SB203580 alone, (3) 5-HT + SB203580, and (4) Veh alone. Example responses are illustrated in Figure 4*B1*, and summary data are presented in Figure 4*B2* . 5-HT led to an 18 ± 12% (*n* = 11) change in p-p38 MAPK at 60 min after treatment, but a similar change (23 ± 12%, *n* = 11) was observed in the group that received the combination of 5-HT + SB203580. A one-way ANOVA found no significant overall effect of the treatments (*F*_(3,40)_ = 1.102, *p* = 0.36)^h^. Thus, SB203580 had no effect on 5-HT-induced levels of p-p38 MAPK at 60 min after 5-HT (Fig. 4*B*), which does not support the possibility of an autoinhibitory loop for p38 MAPK. Combined with the findings that U0126 decreased pERK and p-p38 MAPK, the results indicate that 5-HT may induce separate, but similar, MAPKK pathways to activate p38 MAPK and ERK.

### p38 MAPK phosphorylation at 45 min after onset of 5-HT treatment

Philips et al. (2007; 2013b) found that two tail shocks to *Aplysia* could induce long-term sensitization of a withdrawal reflex but only when spaced by 45 min. No LTM was observed with an ISI that was too short (15 min) or too long (60 min). Therefore, it is important to examine the dynamics of p-p38 MAPK in response to spaced and massed stimuli to obtain insights into whether the delayed activation of p-p38 MAPK by 5-HT is involved in determining the efficacy of different protocols to induce LTM.

**Figure 5.**
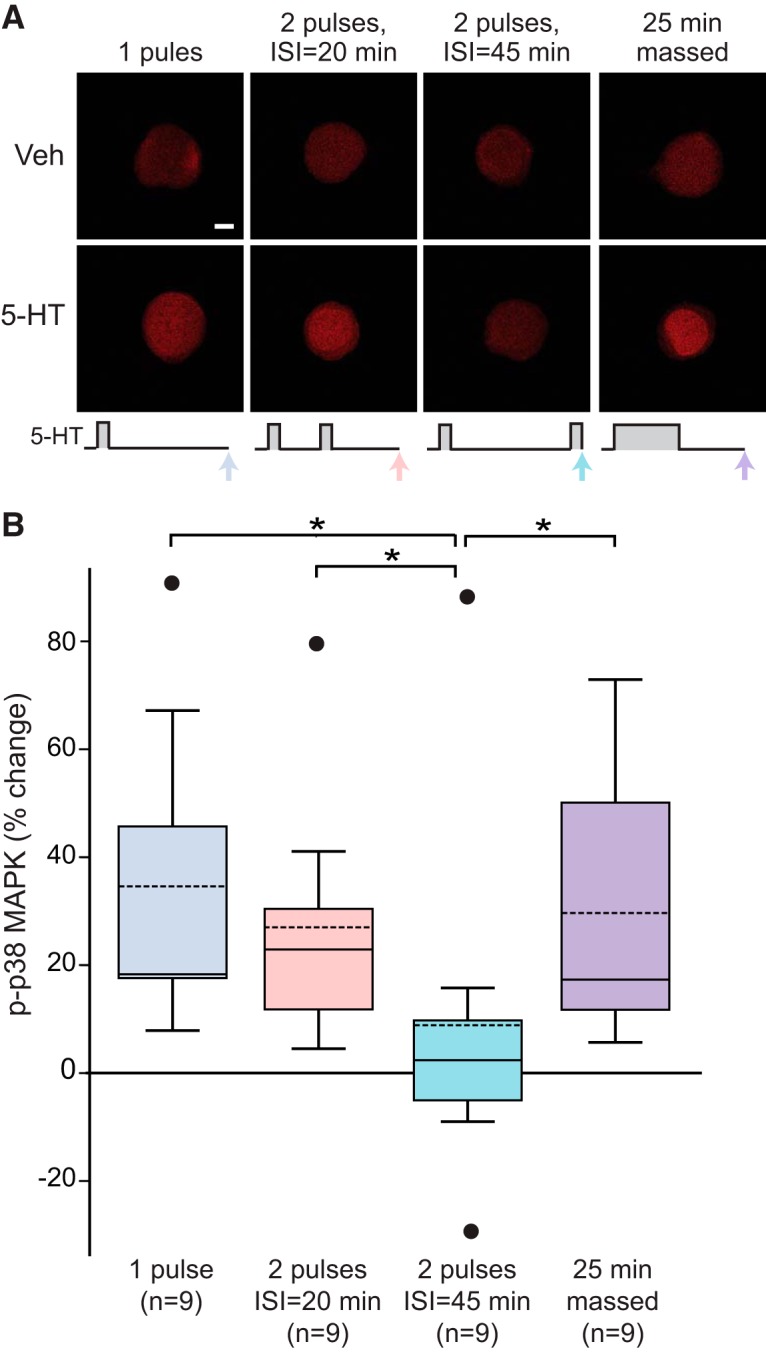
p-p38 MAPK 45-50 min after onset of different stimulus protocols. ***A***, Representative confocal images of p-p38 MAPK immunofluorescence in SNs at 45-50 min after onset of 5-HT. ***B***, Summary data. Levels of p-p38 MAPK was measured at 45 min after onset of a single 25-min 5-HT pulse, two 5-min duration pulses of 5-HT with ISI of 20 min, or one 5-min duration pulse of 5-HT. p-p38 MAPK was measured at 50 min after onset of two pulses of 5-HT with an ISI of 45 min. The last group produced less p-p38 MAPK than any of three former groups. Scale bar, 20 μm. Significant differences are indicated by * for *p* < 0.05.

Immunofluorescence of isolated SNs was used to quantify p-p38 MAPK after four different 5-HT treatments: a 25-min continuous application of 5-HT (i.e., massed), two 5-min applications separated by 20 min or 45 min (i.e., spaced), and a single 5-min application (control group). An ISI of 20 min is commonly used in 5-HT protocols to induce LTF (Martin et al., 1997; Sharma et al., 2003; Zhang et al., 2012). Because pERK increases ∼45 min after the onset of 5-HT (Philips et al., 2013b and confirmed herein) and two stimuli separated by 45 min lead to LTM (Philips et al., 2007), it is important to quantify p-p38 MAPK 45 min after 5-HT onset and compare the effects of different ISIs (20 vs 45 min). A stimulus protocol that significantly decreases p-p38 MAPK would plausibly be effective in induction of LTF. Four treatment groups were examined: (1) one pulse of 5 min 5-HT and fixed at 45 min after onset of 5-HT; (2) two 5-min pulses with ISI of 20 min and fixed 20 min after 5-HT treatment ended; (3) one 25 min pulse as used by Mauelshagen et al. (1998) and fixed 20 min after treatment ended; and (4) two 5-min pulses with ISI of 45 min and fixed immediately after treatment ended. In the first three groups, p-p38 MAPK was measured at 45 min after 5-HT onset, whereas p-p38 MAPK was measured 50 min after 5-HT onset in the fourth group. Example responses are illustrated in Figure 5*A*, and summary data are presented in Figure 5*B*; 45 min after 5-HT onset, two pulses with an ISI of 20 min led to a 27 ± 7.6% increase in p-p38 MAPK (*n* = 9), whereas the increase from 25 min 5-HT was 30 ± 8.0% (*n* = 9). In contrast, two pulses with an ISI of 45 min only increased p-p38 MAPK by 9 ± 11% (*n* = 9). Consistent with data of Figure 2, one pulse of 5-HT produced a substantial increase (35 ± 9.2%, *n* = 9) in levels of p-p38 MAPK 45 min after onset.

Kruskal--Wallis one-way ANOVA on ranks revealed a significant overall effect of the treatments (*H_3_* = 9.531, *p* < 0.05)^i^. Subsequent pairwise comparisons (Student--Newman--Keuls) revealed that the group receiving two pulses of 5-HT with ISI of 45 min was significantly different from the other groups (vs one-pulse group: *q* = 4.018, *p* < 0.05; vs two pulses with ISI of 20 min: *q* = 4.368, *p* < 0.05; vs 25 min 5-HT: *q* = 6.181, *p* < 0.05)^i^. No significant difference was detected among the other groups.

In SNs treated with two pulses of 5-HT with an ISI of 45 min, p-p38 MAPK remained around the control level ∼50 min after onset of 5-HT. This low level of p-p38 MAPK immediately after the 45-min pulse might facilitate the induction of LTF. In contrast, the high level of p-p38 MAPK at 45 min after the massed protocol may contribute to a state that is not permissive for LTF.

### A computational model predicted roles of pERK and p-p38 MAPK in determining the efficacy of spaced 5-HT protocols to induce LTF

**Figure 6.**
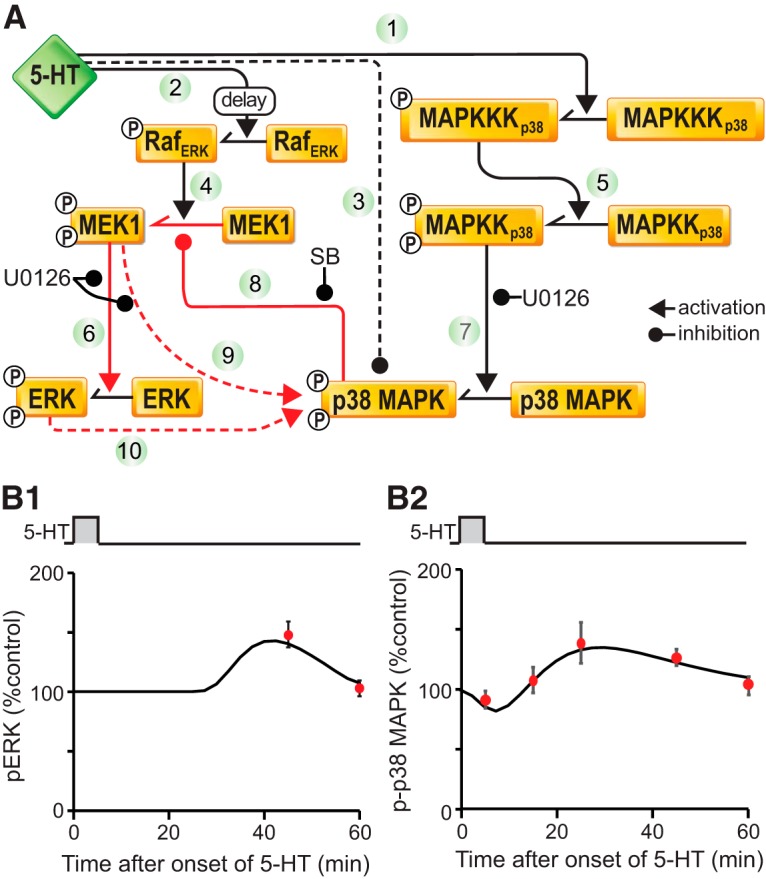
Computational simulation of ERK and p38 MAPK phosphorylation after different 5-HT treatments. ***A***, Schematic of the model of the ERK and p38 MAPK signaling pathways. Key pathways are labelled by numbers. ***B***, Dynamics of pERK (***B1***) and p-p38 MAPK (***B2***) after one pulse of 5-HT. The simulations (black traces) are qualitatively similar to the empirical data (red circles) collected in the present study.

To help provide insights into the possible consequences of the dynamics of p38 MAPK and ERK on the induction of LTF, a computational model was developed (Fig. 6*A* and Materials and Methods). A key model assumption is that 5-HT inhibits and activates p-p38 MAPK through two independent pathways. The inhibitory pathway acts rapidly and predominates during exposure to 5-HT. The stimulatory pathway is MEK dependent and activates slowly and becomes predominant after 5-HT washout. Model parameters were initially constrained so that a single 5-min pulse of 5-HT elicited a delayed phosphorylation of ERK (Fig. 6*B1*) as well as a transient inhibition, followed by a delayed phosphorylation of p38 MAPK (Fig. 6*B2*). The empirical data used to constrain the model parameters were: the levels of pERK at 45 and 60 min after onset of 5-HT (Fig. 6*B1*) and of p-p38 MAPK at 5, 15, 25, 45, and 60 min after onset of 5-HT (Fig. 6*B2*). These data were obtained from the experiments of Figures 1-5, except that pERK at 45 min was measured in additional experiments. The simulation results (black curves) are similar to the empirical data (red circles). The simulation of pERK dynamics (Fig. 6*B1*) is also consistent with the time course measurements by Philips et al. (2013b).

**Figure 7.**
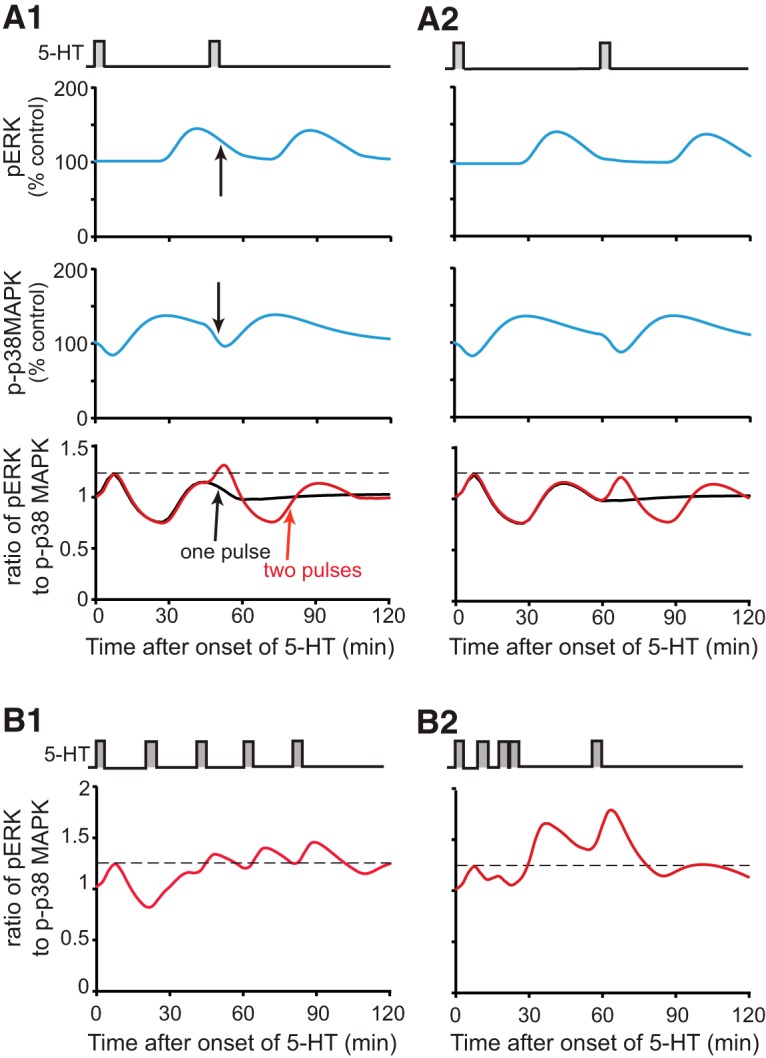
Computational simulation of the ratio of pERK to p-p38 MAPK after two or five pulses of 5-HT. ***A***, Dynamics of pERK and p-p38 MAPK levels, and the ratio of pERK to p-p38 MAPK, for 2 hours after two pulses with ISI of 45 min (***A1***) or 60 min (***A2***). ***B***, Dynamics of the ratio of pERK to p-p38 MAPK for two hours after five pulses with ISIs of the standard protocol (***B1***) or enhanced protocol (***B2***) as compared with the peak ratio of pERK to p-p38 MAPK after one pulse (∼1.2, dashed line).

The model was then used to simulate the time courses of pERK and p-p38 MAPK after two pulses of 5-HT treatment with ISI of 45 min (Fig. 7*A1*). The simulations exhibited a phase shift between the peaks of pERK and p-p38 MAPK. Approximately 50 min after onset of 5-HT, when pERK remained elevated, p-p38 MAPK approached its control level (Fig. 7*A1*, black arrows), consistent with empirical results in Figure 5. To quantify the differential effects of two-pulse 5-HT protocols with different ISIs, the time course of the ratio of pERK to p-p38 MAPK was simulated for 2 h after onset of 5-HT (Fig. 7*A1*, red curve), and compared with the time course of the ratio of pERK to p-p38 MAPK from one pulse (Fig. 7*A1*, black curve). The peak ratio of pERK to p-p38 MAPK after one pulse occurred immediately after the end of 5-HT, due to the decrease of p-p38 MAPK when pERK remained at basal level. The peak ratio of pERK to p-p38 MAPK after one pulse was ∼1.2 (dashed line). The peak ratio of pERK to p-p38 MAPK after two pulses of 5-HT treatment with ISI of 45 min was ∼1.3, occurring at about 50 min. It is apparent that a second pulse with ISI of 45 min produced a greater peak ratio of pERK to p-p38 MAPK than the first pulse. In contrast, a second pulse with ISI of 60 min failed to produce a greater peak ratio of pERK to p-p38 MAPK than the first pulse (Fig. 7*A2*), and neither did a second pulse with ISI of 20 min (data not shown). Given that ERK phosphorylation is associated with LTF, whereas p38 MAPK phosphorylation is associated with LTD, these results suggest a possible explanation for why the ISI of 45 min is superior in inducing LTM: only with an ISI of 45 min is the peak ratio of pERK to p-p38 MAPK higher than the peak produced by one pulse.

The model in Zhang et al. (2012), which lacked the p38 MAPK pathway, predicted, and empirically validated, a novel enhanced protocol of five 5-min pulses of 5-HT that produced stronger LTF and LTM than the standard protocol commonly used to induce LTF in *Aplysia* (five 5-min pulses of 5-HT, with uniform ISI of 20 min) (Fig. 7*B1*). This enhanced protocol has irregular ISIs (10, 10, 5, 30 min) (Fig. 7*B2*), which help generate a greater overlap of PKA and ERK activities than the standard protocol does. With the current model, the peak ratio of pERK to p-p38 MAPK after the enhanced protocol was ∼1.8 (Fig. 7*B2*), whereas the peak ratio of pERK to p-p38 MAPK after the standard protocol was less, ∼1.4 (Fig. 7*B1*). This greater peak ratio may also contribute to the efficacy of the enhanced protocol.

## Discussion

### Dynamic regulation of p38 MAPK activity

Previously, Guan et al. (2003) hypothesized that the 5-HT-induced inhibition of p38 MAPK activity (Fig. 1, 2, 6*A* , pathway 3) relieved an inhibitory constraint on the induction of LTF. Results from the present study indicate that this previous hypothesis needs to be extended. Our results indicate that 5-HT also elicited a late increase in p38 MAPK activity. This biphasic regulation of p38 MAPK by 5-HT is likely to have profound consequences for the understanding of the induction of LTF at sensorimotor synapses of *Aplysia* and possibly other forms of long-term plasticity in other systems. For example, spaced learning is, in general, superior to massed learning in formation of LTM or LTF (Donovan and Radosevich, 1999; Cepeda et al., 2006; Vlach, 2014; Smolen et al., 2016), but not all spaced learning protocols are efficient in memory formation. Philips et al. (2007) found that in *Aplysia*, two tail shocks with ISI of 45 min form LTM, but shorter (15 min) or longer (60 min) ISIs fail to induce LTM. Zhang et al. (2012) used a computational model to suggest that the amount of overlap between PKA and ERK pathways plays a critical role in determining the efficacy of spaced protocols. Overlap between PKA and ERK activities was greatest when the two-pulse protocol was simulated with ISI of 45 min. Here, we extended this model to include the regulation of p38 MAPK by 5-HT. Data suggest that the dynamics of p38 MAPK and ERK are also important for determining the efficacy of spaced learning, leading us to hypothesize that the ratio of ERK to p38 MAPK activity may contribute to the efficacy of spaced 5-HT protocols. In particular, data indicate that two pulses of 5-HT with an ISI of 45 min, but not 20 or 60 min, induce LTM. Corresponding simulations indicate that an ISI of 45 min increased the ratio of pERK to p-p38 MAPK more than did the protocols with ISI of 20 min or 60 min, or one pulse of 5-HT (Fig. 7*A*). Thus, the 45-min ISI appears to leverage the dynamics of the PKA, ERK, and p38 MAPK pathways to produce superior long-term plasticity. In mammals, p38 MAPK activity is also important for LTD (Bolshakov et al., 2000; Murray and O’Connor, 2003). However, the effects of p38 MAPK in *Aplysia* and mammals differ in that p38 MAPK inhibition does not affect LTP in the rat dentate gyrus (Murray and O’Connor, 2003), whereas it enhances LTF in *Aplysia* (Guan et al., 2003).

### Cross talk between ERK and p38 MAPK pathways

5-HT leads to a delayed activation of ERK, which peaks at about 45 min (Philips et al., 2007). Interestingly, the increase rapidly declines by 60 min. SB203580 inhibits downstream effects of p38 MAPK by binding the ATP-binding pocket of p38 MAPK, therefore preventing p38 MAPK from interacting with substrates (Cuenda and Rousseau, 2007). It was found that SB203580 increased pERK at 60 min after onset of 5-HT, suggesting that p38 MAPK inhibits ERK activation (Fig. 3), possibly through inhibition of MEK (Westermarck et al., 2001; Fig. 6*A*, pathway 8). Indeed, studies in human embryonic fibroblasts suggest that p38 MAPK inhibits the MAPK kinase (MAPKK) isoforms MEK1/2, the upstream activators of ERK (Shaul and Seger, 2007), by enhancing their dephosphorylation (Westermarck et al., 2001; Junttila et al., 2008). Thus, it is possible that p38 MAPK is responsible for the reduction of ERK activity 60 min after 5-HT. A delay evidently exists between the activation of p38 MAPK, ∼45 min after 5-HT onset, and the subsequent decline of ERK, ∼ 60 min after 5-HT onset. This delay suggests that indirect inhibition of ERK activation by p38 MAPK includes one or more relatively slow intermediate molecular steps.

Such inhibition of ERK by p38 MAPK might help to explain the observations of some previous studies. For example, Guan et al. (2002) found that if FMRFa and 5-HT are applied to neurons simultaneously, LTD prevails over LTF. It is possible that the activation of p38 MAPK by FMRFa inhibits ERK, accounting in part for the override of LTF by LTD. Liu et al. (2014) found that treatment with a cancer chemotherapeutic drug doxorubicin (DOX) increases pERK and p-p38 MAPK at the same time in *Aplysia* SNs, but DOX suppresses 5-HT-induced LTF and enhances FMRFa-induced LTD. These results may be, at least partially, due to inhibition of the ERK pathway by p38 MAPK. In the present study, SB203580 did not change the level of p-p38 MAPK itself (Fig. 4*B*), suggesting that p38 MAPK does not induce a self-inhibitory feedback loop after its delayed phosphorylation (Fig. 6*A*, pathways 9 -> 8 -> 9 or pathways 6 -> 10 -> 8 -> 6). We note that our current model and simulations (Figs. 6, 7) do not include other MAPKK or MAPK isoforms (e.g., JNK MAPK isoforms), the activation dynamics of which might influence pERK or p38 MAPK activities. Extant data do not, however, appear to substantially constrain and, thus, allow simulation of these possible modes of cross talk.

The mechanism underlying the delayed increase in p38 MAPK activity (Fig. 2) is unknown. It may be protein synthesis-dependent, similar to the delayed (∼45 min) increase in ERK activity (Philips et al., 2013b). However, protein synthesis dependence cannot be examined with the commonly used protein synthesis inhibitors emetine and anisomycin, because emetine and anisomycin have been reported to activate p38 MAPK (Barros et al., 1997; Kim et al., 2015).

It would also be of interest to examine whether at these later times after 5-HT (∼45 min), CREB2 is phosphorylated by p38 MAPK, as was observed by Guan et al. (2002, 2003) after FMRFa. A delayed phosphorylation of CREB2 might, by displacing CREB1 from the promoter of genes regulated by CREs, limit the amount of transcription underlying the induction of LTF, thus contributing to a “ceiling” on the amplitude of LTF.

Interestingly, the increase in p38 MAPK activity appears dependent on a MAPKK pathway, because it is attenuated by application of U0126 (Fig. 4*A*). However, the details of this pathway remain unclear and need to be investigated in future work. For example, U0126 is considered a highly selective inhibitor of MEK1/2 in mammalian cells and not effective in inhibiting two other MAPKKs, MKK3/6 (Duncia et al., 1998). U0126 suppresses the activity of ERK in *Aplysia* (Sharma et al., 2003). In the present study, U0126 also inhibited the activation of p38 MAPK in *Aplysia* (Fig. 4*A*). This result indicates that if U0126 also selectively inhibits a MEK1/2 analogue in *Aplysia*, p38 MAPK should be activated by MEK1/2 analogue in *Aplysia*, not by an MKK3/6 analogue as observed in mammalian cells (Raingeaud et al., 1996; Shaul and Seger, 2007). Alternatively, the action of U0126 may be less selective in *Aplysia*, inhibiting a MAPKK isoform such as MKK3/6 and, thereby, decreasing p38 MAPK activity. Further investigation is needed to determine whether U0126 can inhibit MKK3 and MKK6 analogues in *Aplysia* SNs, as well as whether such pathways activate p38 MAPK in *Aplysia*.

## Synthesis

Reviewing Editor: Elizabeth Cropper, Mt Sinai Sch Med Decisions are customarily a result of the Reviewing Editor and the peer reviewers coming together and discussing their recommendations until a consensus is reached. When revisions are invited, a fact-based synthesis statement explaining their decision and outlining what is needed to prepare a revision will be listed below. The following reviewer(s) agreed to reveal their identity: Thomas Carew, Thomas Abrams.

Both reviewers agree that this manuscript is important and timely and suggest minor revisions. Below specific comments are included, which should be addressed, if appropriate, with editorial changes to the manuscript. Further experiments are not required.

Reviewer #1

General comments:

This article provides an important characterization of p38 MAPK activation during 2-trial 5-HT training. The results help complete an informative view of the molecular cascades and the required molecular interactions responsible for long-term memory formation. The computational model proposed by the authors, while building upon previous computational models and reflecting experimental observations of both ERK and p38 MAPK activation, fails to include other MAPKs which share upstream activators with ERK and p38 MAPK such as the JNKs, which may alter the time courses or peak activations of the simulations (Figures 6 and 7).

Specific suggestions:

1) The authors may wish to include in the discussion the significance of p38 MAPK in vertebrate learning (see Murray et al., 2003, especially noting differences with the findings of Guan et al., 2003).

2) Whereas a requirement for protein synthesis has been established for the 45 minute ERK activation in Aplysia (e.g. Philips et al., 2013), the authors do not investigate if there is a requirement for protein synthesis in the biphasic p38 MAPK activation.

Reviewer #2

The empirical data presented in this study are supplemented by modeling studies that analyze the combined opposing inputs contributions of ERK and p38 MAPK. These empirical data are very robust, and both the empirical results and the modeling result are quite interesting. There are no substantive concerns about this manuscript. However, there are a number of small issues with the presentation; with minor revisions, the manuscript could be substantially strengthened. Although some of the comments below go into substantial detail about how the logic of the presentation can be improved, each of these modifications could be achieved with the addition of a sentence of two or by changing an axis on a graph. A revised manuscript that addresses these comments should not require re-review.

1. Some of the results are particularly striking and merit more emphasis, particularly in the Abstract and Introduction. For example, the dip in p38 MAPK activity 45 minutes after the first of two 5-HT pulses with a 45 minute separation is quite remarkable, particularly because no such dip is observed with a single 5-HT pulse (Fig. 5). This dip could account for the efficacy of the optimal 45 interval between two sensitizing stimuli described by Philips and colleagues. This finding is worth emphasizing in the Abstract and possibly the Introduction.

2. The effect of the MEK inhibitor U0126 to inhibit p38 activation is anomalous. There are two issues that raise doubts about the interpretation of this result. First, U0126 is usually thought to be specific for the MAPK kinase forms, MEK1/2, that activated ERK1/2, and not those the MAPKK that activates p38. One wonders whether this inhibitor is less selective in Aplysia. Second, p38 MAPK is known to inhibit MEK, and the authors find that the p38 inhibitor SB203580 enhances ERK activation at 60 minutes following a pulse of 5-HT, consistent with this inhibitory effect of p38 on ERK activation by MEK. However, using the same protocol, the authors observe no similar effect of SB203580 on p38 activation, which should occur if p38 were downstream of MEK1/2. Thus, despite the puzzling pharmacology, it is difficult to conclude that the MAPKK that activates p38 is the same form that activates ERK. It is evident that the authors understand this logic, but they do not present it sufficiently clearly. A few instances that are confusing are mentioned here:

(1) Abstract- “This activation [of p38] was blocked by a MAPK kinase (MAPKK) inhibitor, suggesting that similar pathways are involved in activation of ERK and p38 MAPK.”

To many readers, “similar pathways” would be interpreted as meaning the same MAPKK. Yet in the model, the authors include a Raf specific for p38 and a p38 MAPKK. It might be less confusing to term the activator of p38 MAPKK MAPKKK-p38, rather than Raf.

(2) “To investigate whether a Raf/MAPKK pathway is involved in the delayed phosphorylation of p38 MAPK, ... in the presence of the MEK1/2 inhibitor U0126. ” (l. 311-313)

This logic eludes me. If the authors expected U0126 were specific for MEK1/2, they would not have initiated this experiment; p38 MAPKK would have been assumed to be U0126 resistant. Conversely, if the authors had reason to believe U0126 is not specific in Aplysia, one would not use it to test for “a Raf/MAPKK pathway” at least not the pathway that normally activates ERK. (We assume a priori that there is a MAPKK that activates p38, but that it should be distinguishable from the MAPKK that activates ERK.) It might be better to present this U0126 result as a surprising observation that is as yet unexplained.

3. In the simulations, “the dephosphorylation rate of MEK1 ... is assumed to be regulated by p-p38 MAPK.” This is entirely reasonable. However, in the literature there is also evidence that PP2A directly dephosphorylates ERK2. Given the lack of effect of SB203580 on p-p38 levels, and the U0126 inhibition of p38 activation, is it possible that in these neurons, p38 acts to inhibit the ERK pathway by stimulating dephosphorylation of ERK rather than MEK?

4. l. 47. Montarolo et al described LTD with the tetrapeptide FMRFa in 1988, and over the next decade or so, Schacher and colleagues studied the phenomenon in a handful of further studies prior to the Guan et al study. Although Guan et al is certainly highly relevant to cite in the context of p38 and LTD, at least one or two of these earlier studies should be cited here.

5. Index for activation (l. 73 and Results). “no effect on ERK activation (i.e., phosphorylation)” It may not be necessary to mention phosphorylation of ERK as a read out for activation in the Introduction. However, if it is mentioned here, a few words of explanation would be helpful. More importantly, beginning in the first paragraph of Results, this assay is used repeatedly without explanation. Also in Methods, under the heading of Pharmacological Treatment, there are repeated references to measuring phosph-p38 or phospho-ERK with no comments as to the significance of these measurements. For the reader who might know little about the MAPK cascades, it would be helpful to include an introduction to this index of ERK or p38 activation at the outset of Results, and possibly also in Methods. Alternatively, there could be a clear explanation in the Introduction.

6. l. 54. “Guan et al. (2002, 2003) proposed a model in which the two pathways are regulated by different inputs.” It would be helpful to conclude this sentence with “and reciprocally inhibit one another.”

7. l. 75-80. The observation of Philips et al. that ERK activation decays by 60 min after 5-HT exposure is mentioned twice in only 4 sentences. These two comments about return to baseline of ERK phosphorylation should be condensed.

8. l. 287. “increase in pERK” should precede parentheses.

9. l. 334-5. “It is possible that U0126 also inhibits a MAPKK isoform that activates p38 MAPK” “distinct from MEK1/2” should follow “isoform.”

10. “Thus SB203580 might be expected to also increase p-p38 MAPK at 60 min post-onset of 5-HT by breaking this inhibitory loop.” (l. 344-345) It seemed confusing that the authors tested for an increase in p38 MAPK activation by SB203580 at the late time point when there is no longer appreciable p38 activation. This experiment makes sense if we consider that the authors had observed that SB203580 increased p-ERK substantially at 60 min. It would be helpful therefore if the explanation for testing this time point reminded the reader that this was the time when inhibiting p38 was found to enhance ERK activation.

11. l. 408-410 “One is rapid and predominates during exposure to 5-HT. The second is MEK dependent and activates slowly and becomes predominant after 5-HT washout. ” This would read better as: The inhibitory pathway acts rapidly and predominates during exposure to 5-HT. The stimulatory pathway is MEK-dependent, and activates slowly and becomes predominant after 5-HT washout.“

12. l. 415. These data were obtained from the experiments of Figs. 1-5, except that pERK at 45 min was measured separately.” Because p-ERK is always measured separately from p-p38, this sentence would be clearer with “was measured in additional experiments.”

13. It is striking that the increase in p-p38 which peaks at 45 minutes after a single 5-HT exposure is not the opposite of the p-ERK increase. Thus, the effect of p38 to inhibit or reverse ERK phosphorylation peaks 15 minutes after the peak of p38 activation. This deserves comment.

14. l. 509-512. “A delayed phosphorylation of CREB2 might, by displacing CREB1 from the promoter of genes regulated by cAMP response elements, limit the amount of transcription underlying the induction of LTF, thus limiting its amplitude.” This effect may account for the bistable effect or ceiling effect on facilitation that has been observed in several studies of LTF in this system, particularly in culture. This possibility should be discussed.

15. Fig. 1 legend. What are the vehicle data in Fig 1A2 and B2 normalized to? Presumably the mean of the vehicle values, which should be stated explicitly in the legend.

16. Fig. 7 legend. The dashed lines should be defined.

17. In a number of figures, effects appear very minor, but the range from 100% to peak is compressed because of the Y axis scale. Figures such as Fig. 1B2 and 4A2 are weakened by the compressed Y axis, which makes the activation of p38 appear to be minimal. In Fig. 2C, the axis could go from 25 to 200% instead of 0 to 300%. The same axis issue reduces the impact of Fig. 6B. The Y axes here are compressed, as they could reasonably go from 75 to 175%. In Fig. 7A, the top curves only range between 100 and 150%, and yet the Y axis is plotted from 0 to 200%.

18. Fig. 6. The early inhibition of p38 by 5-HT should be shown with a broken or doted line, as this is clearly an indirect effect.
